# Vildagliptin preserves the mass and function of pancreatic β cells via the developmental regulation and suppression of oxidative and endoplasmic reticulum stress in a mouse model of diabetes

**DOI:** 10.1111/dom.12005

**Published:** 2012-09-25

**Authors:** S Hamamoto, Y Kanda, M Shimoda, F Tatsumi, K Kohara, K Tawaramoto, M Hashiramoto, K Kaku

**Affiliations:** Division of Diabetes, Endocrinology and Metabolism, Kawasaki Medical SchoolKurashiki, Japan

**Keywords:** β cell, DPP-IV inhibitor, incretins

## Abstract

**Aim:**

We investigated the molecular mechanisms by which vildagliptin preserved pancreatic β cell mass and function.

**Methods:**

Morphological, biochemical and gene expression profiles of the pancreatic islets were investigated in male KK-A*^y^*-TaJcl(KK-A*^y^*) and C57BL/6JJcl (B6) mice aged 8 weeks which received either vildagliptin or a vehicle for 4 weeks.

**Results:**

Body weight, food intake, fasting blood glucose, plasma insulin and active glucagon-like peptide-1 were unchanged with vildagliptin treatment in both mice. In KK-A*^y^* mice treated with vildagliptin, increased plasma triglyceride (TG) level and islet TG content were decreased, insulin sensitivity significantly improved, and the glucose tolerance ameliorated with increases in plasma insulin levels. Furthermore, vildagliptin increased glucose-stimulated insulin secretion, islet insulin content and pancreatic β cell mass in both strains. By vildagliptin, the expression of genes involved in cell differentiation/proliferation was upregulated in both strains, those related to apoptosis, endoplasmic reticulum stress and lipid synthesis was decreased and those related to anti-apoptosis and anti-oxidative stress was upregulated, in KK-A*^y^* mice. The morphological results were consistent with the gene expression profiles.

**Conclusion:**

Vildagliptin increases β cell mass by not only directly affecting cell kinetics but also by indirectly reducing cell apoptosis, oxidative stress and endoplasmic reticulum stress in diabetic mice.

## Introduction

Type 2 diabetes mellitus is a chronic and progressive disease, the underlying pathology of which comprises abnormal insulin secretion caused by impaired β-cell function as well as insulin resistance in target tissues 1. The β-cell function is generally decreased by more than 50% by the time an individual is diagnosed with type 2 diabetes 2,3. To maintain adequate long-term glycaemic control, preventive intervention against the progression of pancreatic β-cell dysfunction is most crucial. Although some anti-diabetic drugs, which are known to possess a protective effect upon pancreatic β cells, are already available, incretin-related drugs recently received remarkable attention for their potential protective effects on pancreatic β cells.

Two known incretin hormones, glucose-dependent insulinotropic polypeptide (GIP) and glucagon-like peptide (GLP-1), are secreted in response to carbohydrates and lipid intake, respectively, from K-cells in the upper small intestine and the L-cell in the distal gut. Both GLP-1 and GIP stimulate glucose-dependent insulin secretion after meal injection by binding to their G-protein coupled receptors expressed at the surface of pancreatic β cells. They reportedly stimulate β-cell replication 4,5, neogenesis 5, differentiation 6 and inhibit β-cell apoptosis 7,8. GLP-1, but not GIP, inhibits glucagon secretion 9 and reduces the rate of gastric emptying 10. Despite the beneficial actions of GLP-1 and GIP, they are rapidly degraded and inactivated by the enzyme dipeptidyl peptidase-IV (DPP-IV) 11. In addition, stimulation of insulin secretion by GIP is markedly reduced in hyperglycaemic type 2 diabetes patients through a defect in β-cell sensitivity to GIP 12. Therefore, DPP-IV inhibitors and long-acting GLP-1 analogues have emerged as a new class of anti-hyperglycaemic drugs. DPP-IV inhibitors augment endogenous active-GLP-1 and -GIP levels, and exert several beneficial effects including an increase in glucose-dependent insulin secretion, suppression of glucagon secretion 13 and augmentation of the pancreatic β cell mass 14.

In this study, we used obese diabetic KK-A*^y^* mice, treated with vildagliptin, to investigate the molecular mechanism by which DPP-IV inhibitor prevents pancreatic β cell damage. To this end, we investigated β cell mass of these mice immunohistochemically and conducted comprehensive analysis of gene expression in the core area of the pancreatic islets, isolated by laser capture microdissection.

## Materials and Methods

### Animals

Eight-week-old male KK-A*^y^*-TaJcl (KK-A*^y^*) mice and C57BL/6JJcl (B6) control mice were obtained from Clea Japan (Tokyo, Japan). The KK-A*^y^* mice were derived from the B6 mice, so we used the B6 mice as the control. All animals were housed in the animal facility of Kawasaki Medical School on a 12 h : 12 h light–dark cycle. The animals were provided free access to standard feed (MF; Oriental Yeast, Tokyo, Japan) and tap water and were maintained at 25 °C. Body weight (BW) and food intake was monitored weekly from 8 weeks of age. These studies were approved by the Animal Use Committee of Kawasaki Medical School (no 08-081 and 10-059) and were conducted in compliance with the Animal Use Guidelines of the Kawasaki Medical School.

### Intervention Protocol

Eight-week-old KK-A*^y^* mice were divided into the following two groups and were treated for 4 weeks: the vildagliptin group (n = 5) received vildagliptin (50 mg/kg body weight/day, oral), and the control group (n = 5) was administered a vehicle. Normal control B6 mice of the same age were also treated with vildagliptin (50 mg/kg body weight/day, oral). The vildagliptin was provided by Novartis Pharma K.K. (Basel, Swiss) and was prepared as an emulsion in distilled water.

### Blood Biochemical Markers

Blood was collected from the tail vein at 8, 10 and 12 weeks of age after an overnight fast (16 h). Blood glucose was measured immediately, and plasma was stored at −80 °C until assay. Blood glucose was measured via an enzyme electrode method using a Free Style kit (Kissei Pharmaceutical, Nagano, Japan). The concentration of plasma insulin (Insulin ELISA kit; Morinaga Institute of Biological Science, Yokohama, Japan), active-GLP-1(GLP-1(active) ELISA kit; Shibayagi, Gunma, Japan) and glucagon (Glucagon ELISA kit; Yanaihara Institute Inc, Shizuoka, Japan) were determined by ELISA. Plasma TG concentration was measured enzymatically using the Triglyceride E-Test Wako (Wako Pure Chemical Industries, Osaka, Japan).

### Oral Glucose Tolerance Test

At the end of the study, an oral glucose tolerance test (OGTT) was performed after an overnight fast. Either the vehicle or vildagliptin was orally administered. After 15 min, the mice were orally delivered glucose (1 g/kg). Blood samples were collected from the tail vein at 15, 30, 60, 90 and 120 min after glucose administration. The plasma insulin level was measured at all points, and active-GLP-1 was measured only at 15 min.

### Insulin Tolerance Test

At the end of the study, an insulin tolerance test (i.p.ITT) was also performed to determine the sensitivity to insulin. Blood glucose levels were measured before and after insulin loading. After a 4-h fast, the mice were intraperitoneally injected human regular insulin (KK-A*^y^*: 2 units/kg body weight, B6: 0.75 units/kg body weight). Blood samples were collected from the tail vein before and 15, 30, 60, 90 and 120 min after insulin administration, and blood glucose levels were measured.

### Measurement of Triglyceride Content and Insulin Content in Pancreatic Islets

At the end of the study, pancreatic islets were isolated by collagenase digestion, as previously reported 15. Briefly, after clamping the common bile duct at its entrance to the duodenum, 3 ml of ice-cold Hank's balanced salt solution (HBSS) containing 1.5 mg/ml collagenase (Collagenase P, Roche, Basel, Switzerland) and 10% (vol./vol.) newborn calf serum (NBCS) was infused into the bile duct. The excised pancreas was incubated at 37 °C for 19.5 min. HBSS containing 10% (vol./vol.) NBCS was added and centrifuged three times (200 g, 2 min). The resultant pellet was passed through a metal filter, and the filtrate was centrifuged (1000 g, 22 min) using Histopaque-1077 (Sigma, St. Louis, MO, USA). The islets were incubated with RPMI medium (Sigma) containing 10% (vol./vol) foetal calf serum (FCS) overnight at 37 °C in 5% CO_2_. On the following day, size-matched pancreatic islets were collected and stored at −80 °C until use in the TG and insulin measurements.

Isolated 45–60 pancreatic islets were washed twice in PBS. A high-salt buffer (50 µl; 2 mol/l NaCl, 2 mmol/l EDTA and 50 mmol/l sodium phosphate) was added, followed by sonication for 1 min to disrupt the pancreatic islets. After centrifugation at 13 000 g for 5 min, 10 µl of the supernatant were mixed with 10 µl of *t*-butanol and 50 µl of Triton X-100-methyl alcohol (1:1). The TG content in the pancreatic islets was measured using a commercially available kit (Triglyceride, E-Test Wako). For insulin measurement, the islets were dissolved in acid ethanol, and the insulin content was measured by ELISA, as described above.

### Glucose-Stimulated Insulin Secretion from Isolated Pancreatic Islets

Pancreatic islets were isolated as described above and were incubated with RPMI medium (Sigma) containing 10% (vol./vol) FCS overnight at 37 °C in 5% CO_2_. Size-matched pancreatic islets were prepared (5 pancreatic islets/tube) and preincubated in KRB-HEPES buffer (containing 5 mg/ml BSA, pH 7.4, saturated 95% O_2−_ 5% CO_2_, 37 °C, 60 min). The supernatant was replaced with either a 3 or 16.7 mmol/l glucose solution, and the mixture was incubated for an additional 60 min. The supernatant was recovered and stored at −80 °C until it could be used in the insulin assay.

### Immunofluorescence

The pancreas was cut into 4-µm sections. To reduce the background staining intensity, sections were incubated with Tris-buffered saline (TBS, pH 7.6) containing 3% (wt/vol.) BSA for 1 h. After washing in TBS, the sections were incubated with a mixture of primary antibodies (mouse anti-glucagon antibody and rabbit anti-insulin antibody; Santa Cruz Biotechnology, Santa Cruz, CA, USA) at 4 °C for 14 h. After rinsing with TBS, a mixture of the second antibodies (Alexa Fluor 488 donkey anti-rabbit IgG and Alexa Fluor 594 donkey anti-mouse IgG; Invitrogen Corporation, Carlsbad, CA, USA) was added, and the reaction was allowed to proceed at 25 °C for 2 h.

### Immunohistochemistry

If necessary, the sections were heated for 15 min at 90 °C in a microwave oven for antigen retrieval, and endogenous peroxidase activity was blocked by immersion in 3% (vol./vol.) hydrogen peroxidase in methanol for 15 min. After washing in TBS, the sections were incubated with either mouse anti-proliferative cell number antigen (PCNA) monoclonal antibody (Nichirei, Tokyo, Japan), or mouse anti-4-hydroxy-2 nonenal modified protein (4-HNE) monoclonal antibody (25 µg/ml; Japan Institute for the Control of Aging, Shizuoka, Japan), or rabbit Ki67 monoclonal antibody (Epitomics, Burlingame, CA, USA), or rabbit anti-CHOP/GADD153 (Santa Cruz Biotechnology) at 4 °C for 14 h. After rinsing with TBS, an adequate amount of second antibody was added, and the reaction was allowed to proceed at 25 °C for 30 min. After another washing in TBS, they were visualized by reacting with a simple stain diaminobenzidine solution (Nichirei) or Vector Red (Vector Laboratories, Burlingam, CA, USA). The sections were counterstained with haematoxylin.

To investigate cell apoptosis, a TUNEL assay was performed using a colorimetric apoptotic detection system (DeadEnd; Promega, Madison, WI, USA).

### Morphometric analysis

The image analysis software NIH Image (version 1.61; http://rsbweb.nih.gov/ij/) was used to calculate the pancreas area and islet area. Using a total of 15 sections (5 sections from three different areas of the pancreas) for each group of mice, β cell mass was estimated via the following formula: cell mass (mg) = average of islet area per section / average of pancreas area per section × weight of pancreas × β cell ratio. Cells positive for PCNA, Ki67 and CHOP staining were quantified by the presence of a dark brown/red nuclear stain. The 4HNE staining was quantified as the positive brown-stained cell cytoplasm. Observations were made using a minimum of 50 islets and, when quantified, were expressed as a percentage of the total number of islet cells.

### Laser capture microdissection

According to the previously established method 16, frozen 8 µm-thick slices were immediately stained with haematoxillin and subjected to laser capture microdissection (LCM) or stored at −80 °C until staining. After tissue staining, the islets were irradiated with a laser using a PixCell system (Arcturus, Mountain View, CA, USA) using 2 s duration, 7.5 µm laser beam at 95 mV power. The islets were recognized morphologically. The islets, larger than 50 µm in diameter, were identified and captured the peripheral region first from slices, and then dissected the remained the β cell rich core area. By using this method, contamination of non-β cells and exocrine cells into the central core sample was avoided.

### Real-time PCR

RNA samples from the islet core tissue obtained by LCM were extracted using a PicoPure RNA Isolation Kit (PN 1220601; Arcturus). TaqMan Reverse Transcription Regents (N808-0234; Applied Biosystems, Foster, CA, USA) were used for reverse transcription. Random hexamers were used as the primers for cDNA synthesis. The primers were designed using Primer Express that was based on mRNA sequences downloaded from the GenBank nucleotide database and the amplicon length was less than 151 bp (Table S1). We confirmed that the amplicons, the length of which were less than 151 bp, led to effective PCR amplification by increase in the sensitivity.

A reaction mixture was prepared by combining 0.5 µl of sample, 1 µl of 50 nmol/l primers, 5 µl of SYBR Green PCR Master Mix (Applied Biosystems) solution and 3.5 µl of diluent solution. Dissociation curve analysis was performed in all experiments to determine the dissociation temperature, and the size of the PCR products was confirmed using agarose gel electrophoresis. To quantify gene expression, 2^−ΔΔCt^ was calculated using 18S rRNA as an internal control.

### Statistical analysis

All data are shown as the mean ± s.e. A Mann–Whitney *U*-test was used for comparison among multiple groups and a *p* < 0.05 was regarded as significant. Multiple comparisons were performed by repeating the Mann–Whitney *U*-test, followed by correction using the Bonferroni–Holm method. Statistical analysis was performed using StatView version 5(SAS, Cary, NC, USA).

## Results

### The Effect of Vildagliptin Upon Metabolic Parameters

**Figure 1:**
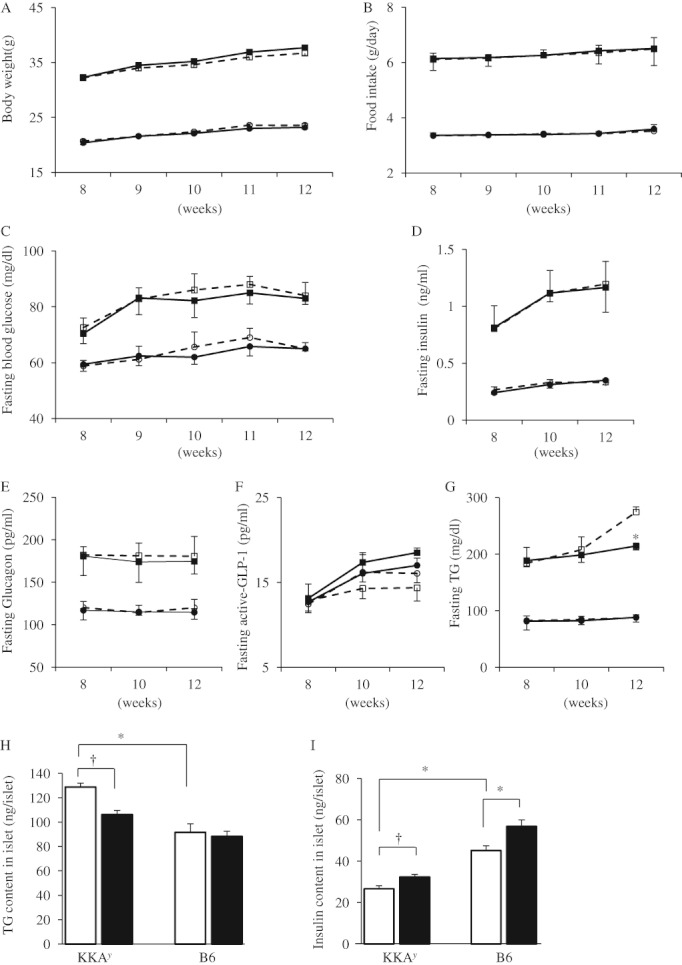
Changes in body weight (A), food intake (B), fasting blood glucose (C), fasting plasma insulin (D), fasting glucagon (E), fasting active-glucagon-like peptide (GLP)-1 (F), fasting triglyceride (TG) (G), islet TG content (H) and islet insulin content (I) in non-diabetic B6 mice and diabetic KK-A*^y^* mice treated with or without vildagliptin (50 mg/kg) for 4 weeks. Squares, KK-A*^y^* mice; circle, B6 mice; black, vildagliptin; and, white, untreated. Data are presented as the mean ± s.e. (n = 5). *p *<* 0.05 vs. control B6 mice; **^†^**p *<* 0.05 vs. control KK-A*^y^* mice.

At the baseline (8 week of age), BW, food intake, fasting blood glucose (FBG), fasting plasma insulin, fasting glucagon, fasting active GLP-1 and TG were similar between the vildagliptin-treated and vildagliptin-untreated groups for either KK-A*^y^* or B6 mice. BW, food intake, FBG, fasting insulin and TG were significantly greater in the KK-A*^y^* mice compared with the B6 mice. Age-dependent increases in BW, food intake, FBG and fasting insulin were shown, but were not affected by the vildagliptin treatment, in both KK-A*^y^* and B6 mice (figure 1A–D). Fasting glucagon and active-GLP-1 were unchanged by vildagliptin administration (figure 1E, F). However, vildagliptin lowered the TG concentration in KK-A*^y^* mice, but not in B6 mice (figure 1G). The TG content in the pancreatic islets, which was significantly greater in KK-A*^y^* than B6 mice, was lowered by vildagliptin only in KK-A*^y^* (figure 1H). At the baseline, the islet insulin content, which was lower at the baseline in KK-A*^y^* than in B6 mice, was significantly increased by vildagliptin treatment in both KK-A*^y^* and B6 mice (figure 1I).

**Figure 2:**
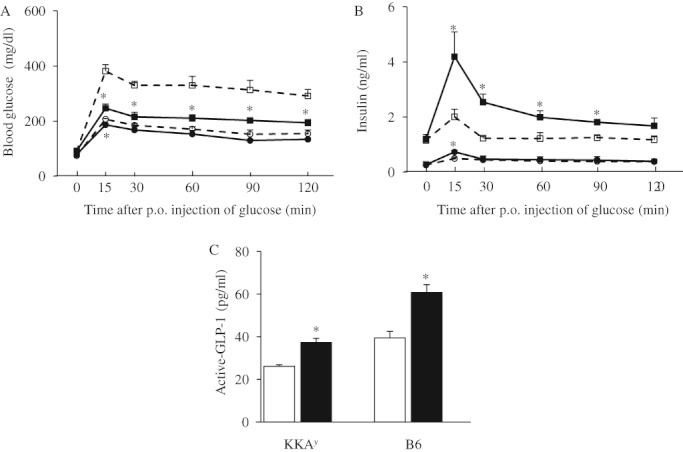
Chronic effect of vildagliptin treatment on blood glucose (A), plasma insulin (B) and active glucagon-like peptide (GLP)-1 at 15 min (C) during oral glucose tolerance test. Squares, KK-A*^y^* mice; circle, B6 mice; black, vildagliptin; and, white, untreated. Data are presented as the mean ± s.e. (n = 5). *p *<* 0.05 vs. control group.

**Figure 3:**
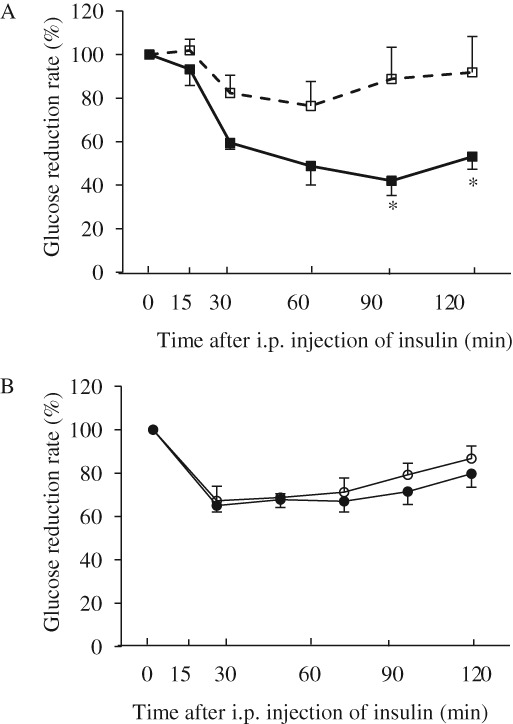
Chronic effect of vildagliptin treatment on insulin sensitivity assessed by insulin tolerance test at 12 weeks of age (A, B). Squares, KK-A*^y^* mice; circle, B6 mice; black, vildagliptin; and, white, untreated. Data are presented as the mean ± s.e. (n = 5). *p *<* 0.05 vs. control group.

Administration of vildagliptin ameliorated glucose tolerance, associated with increased plasma insulin at most of the points of measurement during OGTT, in KK-A*^y^* (figure 2A, B). In B6 mice, a similar improvement by vildagliptin was observed at only 15 min after the glucose challenge. The active form of GLP-1 was significantly higher for the vildagliptin-treated group than for the vildagliptin-untreated group in both strains compared with the control at 15 min following an oral glucose load (figure 2C), which may account in part for the increase in plasma insulin levels at this time point. Finally, the ipITT demonstrated a significant improvement of insulin sensitivity by vildagliptin treatment in KK-A*^y^* mice, but not in B6 mice (figure 3). These results indicated that chronic vildagliptin treatment improved glucose tolerance, insulin sensitivity and lipid profiles in KK-A*^y^* mice but had little or no effect on B6 mice.

### Glucose-Stimulated Insulin Secretion from Isolated Pancreatic Islets

To investigate the background underlying the improved glucose tolerance and insulin sensitivity in mice treated with vildagliptin, we investigated glucose-stimulated insulin secretion from isolated pancreatic islets. Basal insulin secretion measured in the presence of 3 mM glucose was similar between the control and vildagliptin-treated groups in both KK-A*^y^* and B6 mice. The response of insulin secretion to high glucose concentration (16.7 mM) in KK-A*^y^* mice was significantly lower than in B6 mice, but vildagliptin restored insulin secretion in KK-A*^y^* mice. Vildagliptin also significantly enhanced insulin secretion in response to a high glucose concentration in B6 mice (Table 1).

**Table 1:** Glucose-stimulated insulin secretion from pancreatic islets of B6 and KKA*^y^* mice treated with or without vildagliptin

	3 mM (ng/ml/islet)	16.7 mM (ng/ml/islet)	Fold-increase (high GLC/low GLC)
B6 control	0.47 ± 0.07	2.96 ± 0.27	6.63
B6 vildagliptin treated	0.48 ± 0.06	4.34 ± 0.32*	9.43*
KKA^y^ control	0.34 ± 0.04	1.06 ± 0.07*	3.43*
KKA^y^ vildagliptin treated	0.30 ± 0.07	1.81 ± 0.30†	6.42†

Data are presented as the mean ± s.e. (ng/ml/islet, five independent experiments per group).

* p *<* 0.05 vs. control B6 mice.

† p *<* 0.05 vs. control KK-A*^y^* mice.

### Vildagliptin Increased β Cell Mass in Both KK-A*^y^* and B6 Mice

**Figure 4:**
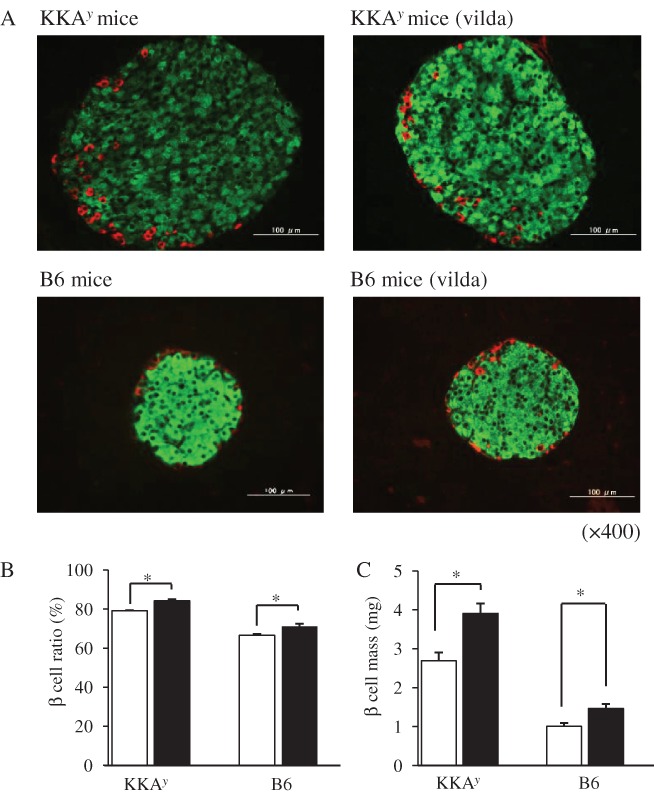
Effect of vildagliptin treatment (4 weeks) on islet morphology. Representative immunofluorescence of insulin (green) and glucagon (red) in pancreatic islet tissue sections from KK-A*^y^* and B6 mice with or without vildagliptin treatment (A). Effect of vildagliptin on β cell ratio (B) and β cell mass (C). Open bars, control group; black bars, vildagliptin group. Data are presented as the mean ± s.e. (n = 5). *p *<* 0.05 vs. control group.

We next employed morphological investigations of isolated pancreatic islets to analyse enhanced glucose-stimulated insulin secretion in vildagliptin-treated mice. Double immunostaining with antibodies against insulin and glucagon showed that distribution of insulin- and glucagon-containing cells were similar between control and vildagliptin-treated groups in bothKK-A*^y^* and B6 mice (figure 4A). Vildagliptin treatment increased the β cell mass by increasing the β cell ratio in KK-A*^y^* mice (figure 4B, C). In B6 mice, the β cell mass and the β cell ratio were also greater in the vildagliptin-treated group than in the vildagliptin-untreated group (figure 4B, C). In contrast, the α cell ratio was decreased by vildagliptin with no change in the α cell mass between the control and the vildagliptin-treated groups in both KK-A*^y^* and B6 mice (data not shown). These results suggested that vildagliptin treatment increased β cell mass but had no effect on the α cell mass for either group of mice.

### Gene Expression Profiles in Pancreatic Islets

To clarify the molecular mechanisms involved in the preventive effect of vildagliptin against pancreatic β cell damage observed elsewhere and above in diabetic model mice, we investigated gene expression profiles in the islet core area by using LCM and real-time PCR (Table 2). The cellular differentiation-promoting genes *Mnx1, Pdx-1, Neurod1, Nkx6-1, MafA* and *Pax6* were significantly upregulated, and the anti-differentiation gene *Hes-1* was downregulated in vildagliptin-treated KK-A*^y^* mice, compared with the controls. Interestingly, upregulation of *Mnx1, Neurod1, Nkx2-2* and *Pax6* genes was also observed in vildagliptin-treated B6 mice. Vildagliptin significantly increased the expression of genes that promote cell proliferation (*Ccnd1* and *Mapk3*) in both B6 and KK-A*^y^* mice. However, the apoptosis-related *Dffb*, *Casp3* and *Casp8* genes, which were expressed at high levels in KK-A*^y^* mice, were significantly downregulated and the anti-apoptotic *Bcl2* gene was upregulated in vildagliptin-treated KK-A*^y^* mice. The oxidative stress-related genes, such as *Cat*, *Gpx1* and *Sod2* were upregulated by vildagliptin in KK-A*^y^* mice, but not in B6 mice. Expression of the endoplasmic reticulum (ER) stress-related *Xbp-1* and *Ddit3* genes were downregulated with vildagliptin treatment in KK-A*^y^*, but not in B6 mice. Expressions of *Fasn*, which was elevated in KK-A*^y^* mice, was significantly reduced by vildagliptin treatment.

**Table 2:** Effect of vildagliptin on gene expression in the islet core area, as measured by LCM and real-time RT-PCR

			B6		KKA^y^	
				
Gene name	Abbreviation	ID	cont	vilda	cont	vilda
Pancreatic hormones						
*InsulinI*	*InsI*	X04725	1	1.06	1	3.30*
*InsulinII*	*InsII*	NM 008384	1	2.72*	1	3.41*
*Glucagon*	*Glucagon*	NM 008100	n.d	n.d	n.d	n.d
*Somatostatin*	*Somatostatin*	NM 009215	1	0.64	1	0.46
Cell differentiation						
*Motor neuron and pancreas homeobox 1*	*Mnx1*	AF153046	1	2.35*	1	2.56*
*Pancreatic and duodenal homeobox 1*	*Pdx-1*	NM 008814	1	1.76	1	2.23*
*Hairy and enhancer of split 1*	*Hes-1*	NM 008235	+	n.d	1	0.32*
*Neurogenic differentiation 1*	*Neurod1*	NM 010894	1	2.85*	1	1.78*
*NK2 transcription factor related, locus2*	*Nkx2-2*	NM 010919	1	1.56*	1	1.24
*NK6 homeobox 1*	*Nkx6-1*	NM 144955	1	1.30	1	4.20*
*v-maf musculoaponeurotic fibrosarcoma oncogene family, protein A*	*Mafa*	AB086961	1	1.27	1	3.36*
*Paired box 6*	*Pax6*	NM 013627	1	4.31*	1	3.32*
Cell proliferation						
*CyclinD1*	*Ccnd1*	NM 007631	1	1.26	1	3.88*
*Mitogen-activated protein kinase3*	*Mapk3*	NM 011952	1	3.56*	1	2.57*
Apoptosis						
*β-cell leukemia/lymphoma 2*	*Bcl2*	NM 177410	n.d	+	1	3.22*
*DNA fragmentation factorbeta subunit*	*Dffb*	AB 009377	n.d	n.d	1	0.28*
*Caspase 3*	*Casp3*	NM 009810	1	0.92	1	0.26*
*Caspase 8*	*Casp8*	MA 077522	1	n.d	1	0.23*
Oxidative stress						
*Catalase*	*Cat*	BC 047126	n.d	n.d	1	4.55*
*Glutathione peroxidase 1*	*Gpx1*	NM 008160	1	0.84	1	4.74*
*Superoxide dismutase 2, mitochondrial*	*Sod2*	NM 013671	1	0.94	1	2.11*
Endoplasmic reticulum stress						
*DNA-damage-inducible transcript 3*	*Ddit3*	X 67083	n.d	n.d	+	n.d*
*X-box binding protein 1*	*Xbp-1*	NM 013842	1	0.78	1	0.13*
Inflammation						
*Tumor necrosis factor*	*Tnf*	NM 013693	+	n.d	1	0.27*
*Intercellular adhesion molecule 1*	*Icam1*	NM 010493	n.d	n.d	+	n.d*
Lipid synthesis						
*Fatty acid synthase*	*Fasn*	NM 007988	1	n.d	1	0.24*

The nondiabetic B6 mice and diabetic KK-A*^y^* mice were treated with or without vildagliptin for 4 weeks. Results (mean of experiments/group) shown are the mRNA levels of vildagliptin-treated mice relative to mRNA of control mice. Cont, control group; vilda, vildagliptin group. The RNA sample for each experiment was collected from the islet core area in one pancreas. When a significant level of mRNA was detected in less than two experiments out of five total experiments, the result was reported as not detected (n.d). +: mRNA was detected in more than or equal to three out of five total experiments, but the ratio was not calculated.

* p < 0.05 vs. control mice.

## Morphological Analysis of Pancreatic Islet Cells

**Figure 5:**
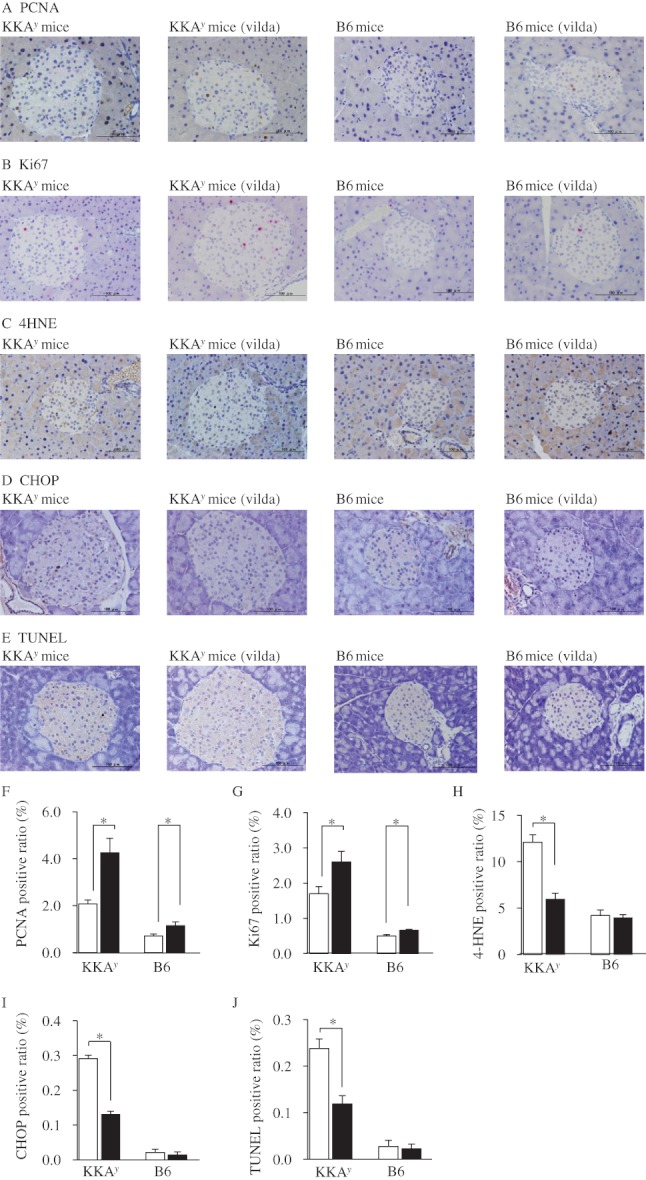
Effect of vildagliptin on proliferation, oxidative stress, endoplasmic reticulum (ER) stress and apoptosis markers in pancreatic islets. Representative immunostaining for proliferation cell nuclear antigen (PCNA) (A), Ki67 (B), 4-hydroxy-2-noneal modified protein (4HNE) (C), CHOP (D) and TUNEL (E) in pancreatic tissue sections. Bars = 100 µm. PCNA, Ki67, 4HNE, CHOP and TUNEL expressions were analysed using a minimum of 50 islets and were expressed as a percentage of the total number of islet cells. Open bars, control group; black bars, vildagliptin group (F–J). Data are presented as the mean ± s.e. (n = 5). *p *<* 0.05 vs. control group.

Morphological examination confirmed and strengthened the results obtained by real-time PCR. Histological sections of the pancreatic islet were stained by antibodies specific for PCNA or Ki67 and the proportion of antibody-positive cells was measured to evaluate the proliferative effect of vildagliptin upon pancreatic islets. Vildagliptin increased the ratio of PCNA positive cells from 2.07% to 4.25% and that of Ki67 positive cells from 1.73% to 2.62% in KK-A*^y^* mice, respectively (0.71% to 1.51% and in 0.50% to 0.66% in B6 mice; figure 5A, B, F, G). Positive staining for 4HNE-modified proteins, a marker of oxidative stress-related lipid peroxidation products, was evident in the cytoplasm of islets from untreated KK-A*^y^* mice (figure 5C), and the ratio of 4HNE positive cells was significantly decreased by vildagliptin treatment only in KK-A*^y^* mice (figure 5H). Cells positive for CHOP, a marker of ER stress 17, in the pancreatic islet were decreased (figure 5D, I), while cellular apoptosis, analysed by TUNEL assay, was suppressed by vildagliptin in only KK-A*^y^* mice (figure 5E, J). These observations are essentially consistent with the results of gene expression analysis and strongly suggest that cell differentiation and proliferation are enhanced while both oxidative and ER stresses are reduced in mice treated with vildagliptin.

## Discussion

In this study, we demonstrated that vildagliptin increased the β cell mass and improved β cell function in diabetic KK-A*^y^* mice, which is consistent with previous reports showing a stimulatory effect of sitagliptin or alogliptin in high fat diet- and streptozotocin-induced type 2 diabetic model mice 13,18. The present results clearly showed that vildagliptin preserved β cell mass in diabetic mice by two different molecular mechanisms.

First, vildagliptin directly enhanced β cell differentiation and proliferation. We showed that vildagliptin increased expression of genes related to cell kinetics in pancreatic islets not only in diabetic KK-A*^y^* but also in non-diabetic B6 mice. These results were also confirmed by the immunohistological staining of pancreatic islets with PCNA and Ki67, suggesting that vildagliptin directly affected β cell differentiation and proliferation, irrespective of the metabolic state of the host animals. The effect of vildagliptin in normoglycaemic animals was also reported previously showing that vildagliptin increased pancreatic β cell mass through enhanced β-cell replication in non-diabetic neonatal Wistar rats 14. Together, these results indicate that the effect of vildagliptin upon expression of genes related to cell differentiation and proliferation is direct and not through the improved glycaemic and/or lipid metabolism.

We also showed that vildagliptin increased the expression of mRNAs including *Mnx1*, *Hes-1* and *Neurod1* in the core area of the pancreatic islets in diabetic KK-A*^y^* and non-diabetic B6 mice. The expression of genes, such as *Mnx1* and *Neurod1*, which play important roles in pancreatic development, had not been studied previously in the adult pancreas. On the other hand, HES1 was activated in the adult pancreas with inflammation, a condition associated with cell regeneration and pancreatic neoplasia 19,20. Furthermore, recent evidence showed that HES1 is involved in determining the fate of adult human β cells *in vitro* 21. The present results suggest that genes associated with an early stage of endocrine pancreas development are expressed in adult mice, and point to the possibility that vildagliptin affects the expression of these genes, thereby regulating β cell proliferation and strongly suggesting the importance of GLP-1 signalling in the protection of pancreatic β cells. Our results are essentially consistent with our previous report showing the increased expression of *Mnx1* and *Neurod1* and the decreased expression of *Hes-1* in the core area of pancreatic islets in diabetic *db/db* and normoglycaemic mice treated with the GLP-1 analogue liraglutide 22.

DPP-IV inhibitor regulates the differentiation, neogenesis and replication of the pancreatic β cells by enabling active incretin hormones to bind their receptors expressed on the cell surface of pancreatic β cells. The binding of GLP-1 and GIP to their receptors activates adenylate cyclase and the cyclic AMP/protein kinase A signalling pathway. Additionally, GLP-1 and GIP activates p42 mitogen-activated protein kinase (MAPK), epidermal growth factor receptor, and the downstream phosphoinositide 3-kinase pathways, including protein kinase B and protein kinase C 4,23–25. Consistent with those results are those showing that modulation of the *Ccnd1* and *Mapk3* gene is involved in the vildagliptin-induced increase in β cell mass, presumably through the MAPK pathway.

The second mechanism involved in the effect of vildagliptin to preserve β cell mass in diabetic mice is the reduction of oxidative stress- and ER stress-related cell apoptosis. It is well established that chronically elevated glucose, lipids and inflammatory cytokines induce not only oxidative stress but also ER stress. We showed in this study that expression of genes related to ER stress and apoptosis were significantly elevated in untreated KKA*^y^* but not in B6 mice. Decreased expression of these genes was associated with improvement of hyperglycaemia and hyperlipidaemia in KKA*^y^* but not in B6 mice chronically treated with vildagliptin. The profile of comprehensive gene expressions in the core area of the pancreatic islets treated with vildagliptin was also supported by immunohistochemical experiments. Thus, we suggest that the suppressive effects of vildagliptin against cell apoptosis, oxidative stress and ER-stress are not direct but, rather, are mediated indirectly secondary to improvement in glycaemic and lipid metabolism.

However, several reports have described how GLP-1 and GIP directly reduce ER stress, oxidative stress and cell apoptosis *in vitro*. Although DPP-IV inhibitors only increase plasma GLP-1 and GIP levels to a high normal or supraphysiological range, the concentrations of GLP-1 and GIP used in these *in vitro* experiments were extremely high. Therefore, the results obtained *in vitro* by using GLP-1 and GIP cannot be directly applied to *in vivo* experiments like those conducted in the present study. In this context, it should be noted that the effect of liraglutide upon ER stress, oxidative stress and cell apoptosis in diabetic *db/db* and normoglycaemic mice are essentially compatible with those observed in this study 22.

One of the important *in vivo* effects of vildagliptin that was observed in this study was improved insulin sensitivity in diabetic KK-A*^y^* mice, which is consistent with reports by Pospisilik and co-investigators, showing that long-term treatment with DPP-IV inhibitor improved insulin sensitivity through reducing hepatic glucose output and enhancing peripheral glucose uptake in VDF Zucker rats 26. Those studies also showed that physiological levels of GLP-1 increased hepatic glucose uptake in a conscious dog 27. Such a beneficial effect of DPP-IV inhibitors upon *in vivo* insulin sensitivity is mediated secondary to the improvement of glucolipotoxicity as shown in this study, although additional molecular mechanisms are suggested. A recent report described the possibility that DPP- IV, as a novel adipokine, may impair insulin sensitivity in adipocyte, skeletal muscle and smooth muscle via inhibition of insulin-stimulated Akt phosphorylation 28. Duez and co-investigators demonstrated that administration of a DPP-IV inhibitor in mice decreased hepatic glucose production with concentrations of GLP-1 and GIP below the level of measurement sensitivity 29. Since DPP-IV is involved in the degradation and inactivation of a number of peptides other than incretin hormones, it is likely that as-yet unidentified DPP-IV-sensitive substrate(s) may also be involved in the improvement of *in vivo* insulin sensitivity.

Finally, we also found that chronic vildagliptin treatment increased insulin content in KKA*^y^* and B6 mice. In general, the islet insulin content is mainly assessed in comparison with plasma glucose and insulin levels. The increased insulin content by vildagliptin treatment is probably due to a reduced insulin requirement in whole body caused by amelioration of glucose intolerance. Furthermore, the increased β cell ratio and β cell mass observed in this study suggested the possibility that an increase in islet insulin content is secondary to an increase in the β cell number or to an increase in β cell insulin granules. In the context of DPP-IV inhibitor-induced insulin secretion from pancreatic β cells, previous studies reported that GLP-1 stimulated proinsulin gene expression and proinsulin biosynthesis in insulinoma βTC-1 cells 30, the cAMP analogue dibutyryl cAMP stimulated insulin biosynthesis in cultured insulinoma cells (RINm5F) 31, and forskolin-stimulated insulin mRNA levels in normal human islets 32. These results suggested the possibility that DPP-IV inhibitor may stimulate insulin biosynthesis via the intracellular cAMP accumulation by incretins and presumably unknown DPP-IV substrate(s). Taken together, we considered that increase in insulin content by vildagliptin treatment was attributable not only to increase in the β cell numbers but also to increase and/or expansion of insulin granules in addition to a reduced requirement of insulin in whole body.

Furthermore, this study showed that vildagliptin enhanced insulin secretion against high glucose stimulation, which is consistent with the results reported by Reimer showing that glucose-stimulated insulin secretion from pancreatic islets, isolated from DPP-IV inhibitor-treated B6 mice, was increased after 8 weeks of treatment, as compared with that in untreated B6 mice 33.

In conclusion, we demonstrated that DPP-IV inhibitor vildagliptin increases pancreatic β cell mass by directly enhancing cell differentiation/proliferation and indirectly suppressing oxidative/ER stress, secondary to improvement in glucolipotoxicity. Further investigations, such as GLP-1 and GIP signalling in pancreatic β cells, should be expected to fully elucidate the mechanisms underlying the protective effects of incretin upon pancreatic β cells.

## Supporting Information

**Table S1.** Primer sequence.

## References

[1] Taylor SI (1999). Deconstructing type 2 diabetes. Cell.

[2] Butler AE, Janson J, Soeller WC, Butler PC (2003). Increased beta-cell apoptosis prevents adaptive increase in beta-cell mass in mouse model of type 2 diabetes: evidence for role of islet amyloid formation rather than direct action of amyloid. Diabetes.

[3] Porte D (1991). Banting lecture 1990. Beta-cells in type II diabetes mellitus. Diabetes.

[4] Friedrichsen BN, Neubauer N, Lee YC (2006). Stimulation of pancreatic beta-cell replication by incretins involves transcriptional induction of cyclin D1 via multiple signalling pathways. J Endocrinol.

[5] Xu G, Stoffers DA, Habener JF, Bonner-Weir S (1999). Exendin-4 stimulates both beta-cell replication and neogenesis, resulting in increased beta-cell mass and improved glucose tolerance in diabetic rats. Diabetes.

[6] Hui H, Wright C, Perfetti R (2001). Glucagon-like peptide 1 induces differentiation of islet duodenal homeobox-1-positive pancreatic ductal cells into insulin-secreting cells. Diabetes.

[7] Widenmaier SB, Kim SJ, Yang GK (2010). A GIP receptor agonist exhibits beta-cell anti-apoptotic actions in rat models of diabetes resulting in improved beta-cell function and glycemic control. PLoS One.

[8] Li Y, Hansotia T, Yusta B, Ris F, Halban PA, Drucker DJ (2003). Glucagon-like peptide-1 receptor signaling modulates beta cell apoptosis. J Biol Chem.

[9] De Marinis YZ, Salehi A, Ward CE (2010). GLP-1 inhibits and adrenaline stimulates glucagon release by differential modulation of N- and L-type Ca2+ channel-dependent exocytosis. Cell Metab.

[10] Naslund E, Gutniak M, Skogar S, Rossner S, Hellstrom PM (1998). Glucagon-like peptide 1 increases the period of postprandial satiety and slows gastric emptying in obese men. Am J Clin Nutr.

[11] Lambeir AM, Durinx C, Scharpe S, De Meester I (2003). Dipeptidyl-peptidase IV from bench to bedside: an update on structural properties, functions, and clinical aspects of the enzyme DPP IV. Crit Rev Clin Lab Sci.

[12] Nauck MA, Heimesaat MM, Orskov C, Holst JJ, Ebert R, Creutzfeldt W (1993). Preserved incretin activity of glucagon-like peptide 1 [7–36 amide] but not of synthetic human gastric inhibitory polypeptide in patients with type-2 diabetes mellitus. J Clin Invest.

[13] Mu J, Woods J, Zhou YP (2006). Chronic inhibition of dipeptidyl peptidase-4 with a sitagliptin analog preserves pancreatic beta-cell mass and function in a rodent model of type 2 diabetes. Diabetes.

[14] Duttaroy A, Voelker F, Merriam K (2011). The DPP-4 inhibitor vildagliptin increases pancreatic beta cell mass in neonatal rats. Eur J Pharmacol.

[15] Kitamura T, Kido Y, Nef S, Merenmies J, Parada LF, Accili D (2001). Preserved pancreatic beta-cell development and function in mice lacking the insulin receptor-related receptor. Mol Cell Biol.

[16] Kanda Y, Shimoda M, Hamamoto S (2010). Molecular mechanism by which pioglitazone preserves pancreatic beta-cells in obese diabetic mice: evidence for acute and chronic actions as a PPARgamma agonist. Am J Physiol Endocrinol Metab.

[17] Yusta B, Baggio LL, Estall JL (2006). GLP-1 receptor activation improves beta cell function and survival following induction of endoplasmic reticulum stress. Cell Metab.

[18] Zhang X, Wang Z, Huang Y, Wang J (2011). Effects of chronic administration of alogliptin on the development of diabetes and beta-cell function in high fat diet/streptozotocin diabetic mice. Diabetes Obes Metab.

[19] Jensen JN, Cameron E, Garay MV, Starkey TW, Gianani R, Jensen J (2005). Recapitulation of elements of embryonic development in adult mouse pancreatic regeneration. Gastroenterology.

[20] Miyamoto Y, Maitra A, Ghosh B (2003). Notch mediates TGF alpha-induced changes in epithelial differentiation during pancreatic tumorigenesis. Cancer Cell.

[21] Bar Y, Russ HA, Knoller S, Ouziel-Yahalom L, Efrat S (2008). HES-1 is involved in adaptation of adult human beta-cells to proliferation in vitro. Diabetes.

[22] Shimoda M, Kanda Y, Hamamoto S (2011). The human glucagon-like peptide-1 analogue liraglutide preserves pancreatic beta cells via regulation of cell kinetics and suppression of oxidative and endoplasmic reticulum stress in a mouse model of diabetes. Diabetologia.

[23] Buteau J, Foisy S, Rhodes CJ, Carpenter L, Biden TJ, Prentki M (2001). Protein kinase Czeta activation mediates glucagon-like peptide-1-induced pancreatic beta-cell proliferation. Diabetes.

[24] Buteau J, Roduit R, Susini S, Prentki M (1999). Glucagon-like peptide-1 promotes DNA synthesis, activates phosphatidylinositol 3-kinase and increases transcription factor pancreatic and duodenal homeobox gene 1 (PDX-1) DNA binding activity in beta (INS-1)-cells. Diabetologia.

[25] Buteau J, Foisy S, Joly E, Prentki M (2003). Glucagon-like peptide 1 induces pancreatic beta-cell proliferation via transactivation of the epidermal growth factor receptor. Diabetes.

[26] Pospisilik JA, Stafford SG, Demuth HU, McIntosh CH, Pederson RA (2002). Long-term treatment with dipeptidyl peptidase IV inhibitor improves hepatic and peripheral insulin sensitivity in the VDF Zucker rat: a euglycemic-hyperinsulinemic clamp study. Diabetes.

[27] Dardevet D, Moore MC, DiCostanzo CA (2005). Insulin secretion-independent effects of GLP-1 on canine liver glucose metabolism do not involve portal vein GLP-1 receptors. Am J Physiol Gastrointest Liver Physiol.

[28] Lamers D, Famulla S, Wronkowitz N (2011). Dipeptidyl peptidase 4 is a novel adipokine potentially linking obesity to the metabolic syndrome. Diabetes.

[29] Duez H, Smith AC, Xiao C (2009). Acute dipeptidyl peptidase-4 inhibition rapidly enhances insulin-mediated suppression of endogenous glucose production in mice. Endocrinology.

[30] Fehmann HC, Habener J (1992). Insulinotropic hormone glucagon-like peptide (7-37) stimulation of proinsulin gene expression and proinsulin biosynthesis in insulinoma betaTC-1 cells. Endocrinology.

[31] Dobs AS, Broussolle C, Lane MD (1989). Regulation of insulin synthesis in an insulin-producting cell line (RINm5F): long-term experiments. In Vitro Cell Dev Biol.

[32] Hammonds P, Schofield PN, Ashcroft SJ, Sutton R, Gray DW (1987). Regulation and specificity of glucose-stimulated insulin gene expression in human islets of Langerhans. FEBS Lett.

[33] Reimer MK, Holst JJ, Ahren B (2002). Long-term inhibition of dipeptidyl peptidase IV improves glucose tolerance and preserves islet function in mice. Eur J Endocrinol.

