# Supplementary material for: Vildagliptin preserves the mass and function of pancreatic β cells via the developmental regulation and suppression of oxidative and endoplasmic reticulum stress in a mouse model of diabetes
Source: Diabetes Obes Metab. 2012 Sep 25;15(2):153–63. doi: 10.1111/dom.12005 (PMC3558804; doi:10.1111/dom.12005)
Supplement: Supplementary file 1 [file dom0015-0153-SD1.docx]

**Supplemental Table.1** Primer sequence

| Abbreviation | Primer sequence | | size |
| --- | --- | --- | --- |
|  | Forward sequence | Reverse sequence |  |
| *18S rRNA* | GCGCTTCCTTACCTGGTTGAT | GCCATTCGCAGTTTCACTGTAC | 108 |
| *InsⅠ* | CCCTTAGTGACCAGCTATAATCAGA | ACCACAAAGATGCTGTTTGACAA | 150 |
| *InsⅡ* | CTGCTGGCCCTGCTCTTC | AACCACAAAGGTGCTGCTTGA | 70 |
| *Glucagon* | GGCACATTCACCAGCGACTA | TCATCAACCACTGCACAAAATCT | 73 |
| *Somatostatin* | CTCTCCCCCAAACCCCATAT | TTTCTAATGCAGGGTCAAGTTGAG | 71 |
| *Mnx1* | CGAGACTCAGGTGAAGATTTGGT | TGCTCTTTGGCCTTTTTGCT | 72 |
| *Pdx-1* | CGGCTGAGCAAGCTAAGGTT | TGGAAGAAGCGCTCTCTTTGA | 71 |
| *Hes-1* | TCCCGGTCTACACCAGCAA | AGCGAGGAGCCACTGGAA | 73 |
| *Neurod1* | AGGAACACGAGGCAGACAAGA | CTCCCCCGTTTCTCAGAGAGT | 78 |
| *Nkx2-2* | CACAGGTCAAGATCTGGTTCCA | GCGTCACCTCCATACCTTTCTC | 78 |
| *Nkx6-1* | CAAACCTCTGGACCCGAACTC | GCTGCCACCGCTCGATT | 76 |
| *Mafa* | CCAGCTGGTATCCATGTCC | TTCTGTTTCAGTCGGATGACC | 90 |
| *Pax6* | CAACCTGGCTAGCGAAAAGC | CCCGTTCAACATCCTTAGTTTATCA | 70 |
| *Ccnd1* | TCGTGGCCTCTAAGATGAAGGA | CCTCGGGCCGGATAGAGTT | 84 |
| *Mapk3* | CCTGCTGGACCGGATGTTA | TGAGCCAGCGCTTCCTCTAC | 66 |
| *Bcl2* | CGCTGCGGTGCTCTTGA | TCACACTCCGGCTTCACTGA | 71 |
| *Dffb* | GCACGGCAACTGCTGTCA | GAAGATCGGCCAGCAGCTT | 61 |
| *Casp3* | CTGGACTGTGGCATTGAGACA | CAGCCTCCACCGGTATCTTC | 73 |
| *Casp8* | GAGATCCTGTGAATGGAACCTGGTA | CACGCCAGTCAGGATGCTAAGA | 101 |
| *Cat* | GCTGAGAAGCCTAAGAACGCAAT | CCCTTCGCAGCCATGTG | 65 |
| *Gpx1* | CCTTGCCAACACCCAGTGA | CCGGAGACCAAATGATGTACTTG | 69 |
| *Sod2* | TTAACGCGCAGATCATGCA | GGTGGCGTTGAGATTGTTCA | 71 |
| *Ddit3* | GGAGCCAGGGCCAACAG | GCCATAGAACTCTGACTGGAATCTG | 75 |
| *Xbp-1* | TTCATGAATGGCCCTTAGCAT | AAAACAAGCCCCCTCAGGTT | 74 |
| *Tnf* | TGATCCGCGACGTGGAA | ACCGCCTGGAGTTCTGGAA | 72 |
| *Icam1* | TCGGAAGGGAGCCAAGTAACT | CGACGCCGCTCAGAAGAA | 73 |
| *Fasn* | CCTGGATAGCATTCCGAACCT | GCACATCTCGAAGGCTACACA | 121 |

Size: Amplicon size (bp)
